# Brain volume estimation from post-mortem newborn and fetal MRI

**DOI:** 10.1016/j.nicl.2014.10.007

**Published:** 2014-10-23

**Authors:** Eliza Orasanu, Andrew Melbourne, M. Jorge Cardoso, Marc Modat, Andrew M. Taylor, Sudhin Thayyil, Sebastien Ourselin

**Affiliations:** a Translational Imaging Group, Centre for Medical Image Computing (CMIC), University College London, UK; b Centre for Cardiovascular Imaging, Institute of Cardiovascular Science, University College London, UK; c Perinatal Neurology and Neonatology, Imperial College London, UK

**Keywords:** CI, confidence interval, CSF, cerebrospinal fluid, EM, expectation maximization, GA, gestational age, GW, gestational weeks, MaRIAS, Magnetic Resonance Imaging Autopsy Study, MRI, magnetic resonance imaging, Post-mortem MRI, Newborn, Fetus, Brain volumes, Autopsy, Cerebrum

## Abstract

**Objective:**

Minimally invasive autopsy using post-mortem magnetic resonance imaging (MRI) is a valid alternative to conventional autopsy in fetuses and infants. Estimation of brain weight is an integral part of autopsy, but manual segmentation of organ volumes on MRI is labor intensive and prone to errors, therefore unsuitable for routine clinical practice. In this paper we aim to show that volumetric measurements of the post-mortem fetal and neonatal brain can be accurately estimated using semi-automatic techniques and a high correlation can be found with the weights measured from conventional autopsy results.

**Methods:**

The brains of 17 newborn subjects, part of Magnetic Resonance Imaging Autopsy Study (MaRIAS), were segmented from post-mortem MR images into cerebrum, cerebellum and brainstem using a publicly available neonate brain atlas and semi-automatic segmentation algorithm. The results of the segmentation were averaged to create a new atlas, which was then used for the automated atlas-based segmentation of 17 MaRIAS fetus subjects. As validation, we manually segmented the MR images from 8 subjects of each cohort and compared them with the automatic ones. The semi-automatic estimation of cerebrum weight was compared with the results of the conventional autopsy.

**Results:**

The Dice overlaps between the manual and automatic segmentations are 0.991 and 0.992 for cerebrum, 0.873 and 0.888 for cerebellum and 0.819 and 0.815 for brainstem, for newborns and fetuses, respectively. Excellent agreement was obtained between the estimated MR weights and autopsy gold standard ones: mean absolute difference of 5 g and 2% maximum error for the fetus cohort and mean absolute difference of 20 g and 11% maximum error for the newborn one.

**Conclusions:**

The high correlation between the obtained segmentation and autopsy weights strengthens the idea of using post-mortem MRI as an alternative for conventional autopsy of the brain.

## Highlights

• We segment the cerebrum of 17 newborns and 17 fetuses from postmortem MR.
• There is high correlation between the autopsy and MR segmentation brain volumes.
• Postmortem MR can be a non-invasive alternative to conventional autopsy of infants.

## 1 Introduction

The loss of a fetus, a baby or a child is traumatizing and devastating for a parent. Knowing the exact reason why their child died and if there is any risk for further pregnancies or existing children is comforting and can help parents cope with their loss. Performing autopsy is important for establishing the cause of death and for the progress in medicine and research. In about 14–46% of perinatal and infant post-mortem examinations, information is found beyond what was known prior to the examination, affecting the counseling and estimate of recurrence risk (Thayyil et al., 2011). Many studies have previously shown that there is low concordance between the pre-mortem and post-mortem diagnosis (Ornelas-Aguirre et al., 2003; Combes et al., 2004), sustaining the need for autopsies to be performed. Over the past decade, the consent rate for autopsy in the newborns has been less than 20% and less than 50% in stillborns in the United Kingdom (Centre for Maternal and Child Enquiries (CMACE), 2011). The main reasons of parental refusal are religion, the invasive nature of the autopsy and delay of the funeral. The usual brain autopsy practice consists of the removal and fixture of the brain before dissection, a process that can take up to 3 weeks (Thayyil et al., 2011). Even when adequately fixated, the high water content of the immature brain makes its handling difficult (Huisman, 2004). Parents usually request that all organs are replaced before the funeral, which means that the brain tissue has to be examined following a suboptimal fixation period, making the detailed structural analysis of the developing brain even more challenging (Huisman, 2004).

MRI is a powerful tool that can be used as a post-mortem imaging technique with high accuracy and high level of performance for depicting soft-tissue lesions (Ross et al., 2012). MRI has been proved to be a credible alternative to invasive autopsy mostly in the case of non-suspicious death, with a diagnosis agreement of 87% (Bisset et al., 2002). Griffiths et al. (2003) examined the neuropathology in fetuses and deceased neonates and found a good agreement of the reasons leading to death between MRI and autopsy in 28 out of 32 cases, concluding that MR provides detailed information about all organ systems, except the heart (Griffiths et al., 2003). Cohen et al. (2008) also found a good correlation for detecting brain and spine anomalies, but concluded that it should be combined with autopsy results in most cases for a precise result (Cohen et al., 2008). Breeze et al. (2006) determined kappa values (statistical measures of inter-rater agreement) in order to assess agreement between autopsy and MRI for different organs and found a very high value for the brain (0.83).

Thayyil et al. (2013) suggested the need for a minimally invasive autopsy procedure, using MRI coupled with blood sampling via needle puncture. To the best of our knowledge, this research presents the most extensive database up to now, containing over 400 post-mortem T1, T2 and/or diffusion MRI scans of fetuses and newborns (Thayyil et al., 2013). Specialists in nervous, cardiovascular, pulmonary and abdominal fields were summoned to study the MRI results for a diagnostic of the cause of death in 400 cases, which was also established from conventional autopsy. The agreement between the minimally invasive autopsy and conventional autopsy was of 89.3%, while using MRI alone had an agreement of 55.5%.

Accurate estimation of brain weight is an integral part of autopsy, since any deviation from the normal ranges could be an indicator of pathological change in the organ and therefore helps in establishing the cause of death. Rapid prototyping of organs may be useful also in explaining the pathologies to parents and to the jury in forensic cases (Addison et al., 2014). During conventional autopsy, the cerebellum and brainstem are usually separated from the cerebrum and are weighed separately. Similarly then, it is useful to segment the cerebrum, cerebellum and brainstem from the brain MR images. Manual segmentation of organ volumes on MRI is labor intensive (Breeze et al., 2008) and unsuitable for routine clinical practice.

The aims of this study are to: 1) Compare a new semi-automatic post-mortem segmentation with the manual segmentation for newborns (35–46 weeks equivalent gestational age); 2) Create a new post-mortem brain atlas for newborns (35–46 weeks equivalent gestational age); 3) Compare the results of the fetal cohort post-mortem brain segmentation (29–44 GW) using the new post-mortem brain atlas for newborns versus a publicly available neonatal atlas; and 4) Compare the estimated brain weights obtained from semi-automatic segmentation with post-mortem conventional autopsy ones in both newborns and fetuses. This is the first study, to the best of our knowledge, to segment both fetal and neonate post-mortem brain MRI using semi-automatic techniques.

## 2 Methods

### 2.1 Subjects

We performed pre-autopsy post-mortem cerebral MRI using a 1.5 Tesla Siemens Avanto MR scanner in 17 fetuses (scanned ex utero) and 17 newborns, as a part of the Magnetic Resonance Imaging Autopsy Study (MaRIAS) (Thayyil et al., 2013). The selection of these particular subjects was based on the fact that they presented no major lesions or cerebral hemorrhage and that their scans had higher resolution, better contrast, and no major artifacts. The newborns were mostly term-born and were aged between 35 and 46 weeks (gestational age (GA) added with age after birth). The fetuses had GA between 29 and 44 gestational weeks (GW), all calculated based on the mother's last menstruation date.

### 2.2 MR acquisition

The scans were acquired at Great Ormond Street Hospital for Children (GOSH) and University College London Hospital (both in London, United Kingdom) between March 1st, 2007 and September 30th, 2011 (Thayyil et al., 2013). For this study, we used the 3D T2-weighted Constructive Interference in Steady State (CISS) MR images which have a voxel size of 0.6 × 0.6 × 0.6 mm^3^, acquisition time of 13.5 min, relaxation time (TR) of 9.2 ms, echo time (TE) of 4.6 ms, and flip angle of 70° with 4 signal averages.

### 2.3 Recruitment

The study was approved by GOSH and Institute of Child Health (ICH) research ethics committee (04/Q0508/41) (Thayyil et al., 2011). The standard National Health Service (NHS) consent form (produced by Department of Health) that includes consenting for the use of post-mortem imaging for research was used (Thayyil et al., 2011). Research nurses approached the parents by telephone and if verbal consent was gained for MR, a pre-paid envelope with consent form and information leaflet was sent to the parents before post-mortem MRI was performed (the consenting process has been previously described) (Thayyil et al., 2009). Conventional autopsy was performed according to the guidelines set down by the Royal College of Pathologists (UK) (Thayyil et al., 2011), during which the cerebrum weight was measured.

### 2.4 Image preprocessing

All images underwent bias field correction using the N3 algorithm of FreeSurfer (Sled et al., 1998). This was necessary to minimize the registration error induced by intensity non-uniformity as a result of the MR acquisition. Masks of the intracranial volume for both cohorts were resampled from a publicly available neonate brain atlas (Kuklisova-Murgasova et al., 2011) 1 after a non-rigid registration between each image and the atlas template. All masks were checked and manually corrected to exclude any non-brain tissue that can generate mislabeled voxels in the subsequent segmentation using ITK-SNAP (Yushkevich, et al., 2006).

### 2.5 Newborn brain segmentation

The segmentation pipeline for the newborn brain consisted of two main stages.

In the first stage, we carried out a non-rigid registration of the masked brain images to the template of the publicly available atlas (Kuklisova-Murgasova et al., 2011). The obtained transformation was then used to resample, from this atlas, the corresponding anatomical priors for 4 different areas of the brain: cerebrum, cerebellum, brainstem and cerebrospinal fluid (CSF). The cerebrum class contained both white and gray matter, as it was not possible to segment them separately likely due to the difference between their T1 and T2 values decreasing significantly after death due to brain decay (Thayyil et al., 2012). Using the derived anatomical priors and a neonate specific Expectation-Maximization (EM) segmentation algorithm with prior relaxation and a Markov Random Field to enforce spatial smoothness (AdaPT) (Cardoso et al., 2013) available in NiftySeg^2^, we segmented the brain into the 4 tissue classes/areas mentioned above.

The second stage consisted of combining together the cerebrum and CSF classes. This was necessary since, because of the aforementioned T1 and T2 increasing values post-mortem, the CSF and cerebrum were difficult to separate mainly in the parietal and frontal lobes. However, having this class at the beginning was necessary in order to get a robust segmentation of the cerebellum and brainstem, which, in newborns and fetuses, are surrounded by fluid.

After applying the pipeline, the newborn brains are segmented into three regions: cerebrum plus CSF (including extra axial spaces and ventricles), cerebellum and brainstem.

### 2.6 Post-mortem newborn average atlas

An average image and an average segmentation were created for the newborn cohort using all 17 images. A groupwise average (Ashburner, 2000) was created using NiftyReg^3^ by performing a sequence of registrations with increasing number of degrees of freedom, each time registering the images to a new average space. We performed one rigid (translation and rotation), four affine (translation, rotation, scaling and shearing) (Ourselin et al., 2001) and four non-rigid (free-form deformation model based on cubic B-splines) (Rueckert et al., 1999; Modat et al., 2010) registration steps. For all of the registration steps mentioned we used default parameters. The number of registration steps in a groupwise typically depends on the diversity of the images. Note that after four non-linear registration steps the transformations from the native image spaces to the average image space did not change. The obtained transformations from each native space to the average space were used to propagate the individual segmentations and create an average segmentation for all brain regions mentioned, as well as an average mask.

### 2.7 Fetal brain segmentation

**Fig. 1:**
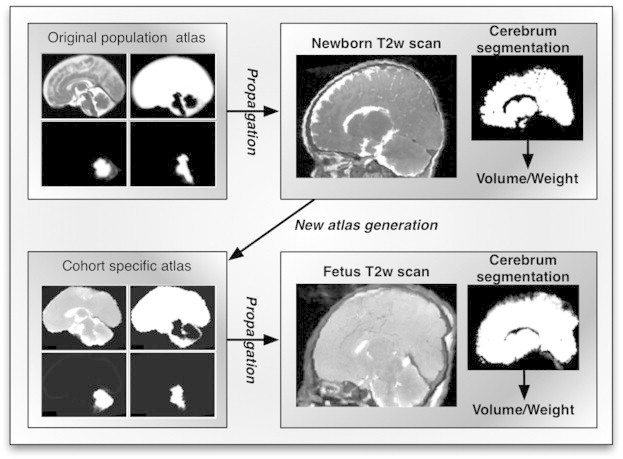
Processing pipeline: A publicly available atlas was used to create the priors for AdaPT segmentation of the newborn cohort. A new atlas was generated from the newborn cohort segmentations and used to create the priors for AdaPT segmentation of the fetus cohort. The cerebrum volumes/weights were computed from the thresholded cerebrum segmentation.

We segmented the fetus cohort into cerebrum plus cerebrospinal fluid, cerebellum and brainstem using the same two-stage segmentation pipeline, the only difference consisting of the anatomical priors used in the first stage. In this case, the fetal brain images were non-rigidly registered to the newly created post-mortem newborn atlas and the transformation obtained was used to resample the anatomical priors from this atlas. We compared the segmentation using this priors to the ones obtained using the available neonate brain atlas (Kuklisova-Murgasova et al., 2011). Using the newly created atlas, rather than the publicly available one, is expected to give a more accurate and precise segmentations because the post-mortem fetus MR images are more similar to the post-mortem newborn ones as a results of undergoing the same changes in tissue contrast that appear after death. The algorithm pipeline describing the segmentation propagation is shown in Fig. 1.

### 2.8 Validation

To perform quantitative evaluation of the automatic segmentations, we (E. Orasanu) manually segmented the MR images from 8 randomly selected subjects using ITK-SNAP (Yushkevich et al., 2006) from each cohort into the three regions of interest: cerebrum and CSF, cerebellum and brainstem. The investigator was blinded to the semi-automatic segmentation results and has substantial image analysis expertise but limited neuroanatomical expertise. We then computed the Dice score overlap (defined here as the number of voxel labels that agree between two images, divided by the average number of voxels with that label in those images), between the automatic and manual segmentations.

### 2.9 Eliminating the fluid from the cerebrum segmentation

The segmentation region of interest for both cohorts is composed of cerebrum and CSF, since they could not be separated. We manually thresholded the segmentation to properly eliminate any fluids that do not contribute to the cerebrum weight obtained during autopsy (Blatter et al., 1995). The fluid threshold values were taken individually for each patient from the bias field corrected images and voxels with higher values than the threshold were excluded from the volume.

### 2.10 Post-mortem cerebrum weights

We computed the volume of the cerebrum as the binary sum of all pixels in the segmentation of the region of interest multiplied by the voxel dimension (provided by the scanner). The cerebrum weight can be estimated by multiplying this volume with a literature-derived density value of the brain of 1.08 g/mL (Breeze et al., 2008). We compared the estimated weights with the autopsy weights, which are available for 15 newborn and 13 fetus subjects.

## 3 Results

### 3.1 Atlas-based brain segmentation

**Fig. 2:**
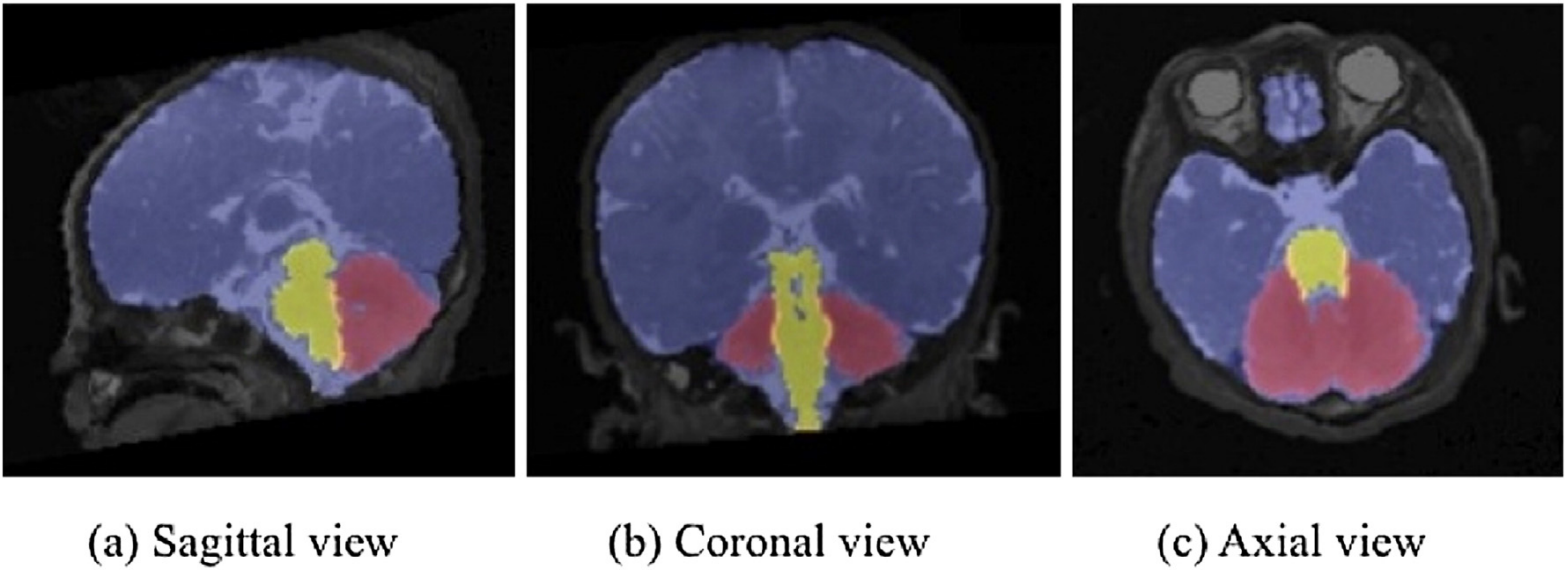
Example segmentation of a MaRIAS newborn (44 week gestation).

An example segmentation is shown in Fig. 2. The average Dice overlap between the automatic and manual segmentations are included in Table 1.

**Fig. 3:**
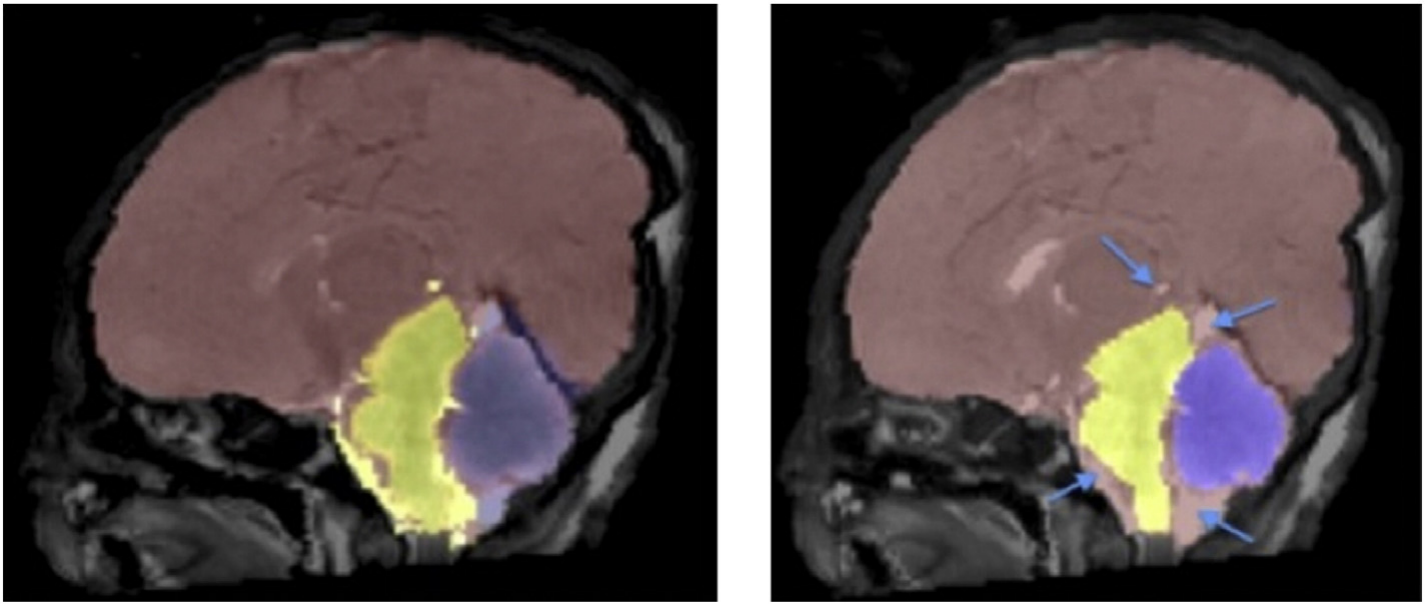
Brain segmentation of MaRIAS fetus of 38 GW using the public atlas (left) and using the MaRIAS newborn atlas (right).

When compared with the manual segmentation, the fetus segmentation obtained using priors derived from the public atlas had lower Dice score values than the ones obtained using the newly created newborn atlas for all three structures. Improvement of the segmentation by using the newly created atlas as template can also be observed in Fig. 3.

### 3.2 Post-mortem cerebrum volumes

**Fig. 4:**
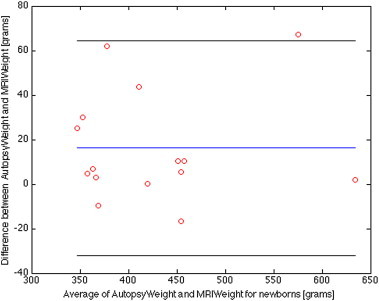
Bland–Altman plot for the MaRIAS newborn cohort showing differences between the conventional autopsy weights of the brain (cerebrum only, no fluid) and the automatic segmented ones from the MR images. The blue line corresponds to the average difference between the autopsy and MRI weight, while the black lines indicate the 95% limits of agreement (average difference ± 1.96 standard deviation of the difference).

**Fig. 5:**
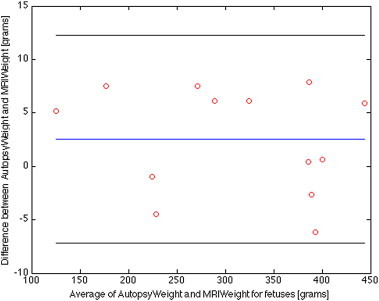
Bland–Altman plot for the MaRIAS fetal cohort showing differences between the conventional autopsy weights of the brain (cerebrum only, no fluid) and the automatically segmented ones from the MR images. The blue line corresponds to the average difference between the autopsy and MRI weight, while the black lines indicate the 95% limits of agreement (average difference ± 1.96 standard deviation of the difference).

We estimated the cerebrum volumes and weights from the segmentation of the MR images for the newborn (Table 2) and fetus (Table 3) cohorts after thresholding to remove CSF. We compared the estimated cerebrum weight for the newborn and fetus cohorts with the available autopsy weights (15 newborns and 13 fetuses). The Bland–Altman plot comparing the MRI weight and autopsy weight are shown for the newborn cohort in Fig. 4 and for the fetal cohort in Fig. 5.

For the newborn cohort, the mean weight estimate by MRI and conventional autopsy was 418 versus 434 g. The mean absolute difference (mean of all the absolute differences between the MRI and conventional autopsy weights) was 20 g and the 95% confidence interval (CI) is [−32, 65] g. For the fetus cohort, the mean weight estimate by MRI and conventional autopsy was 310 versus 312 g. The mean absolute difference was 5 g and the 95% CI is [−7, 12] g.

## 4 Discussions

We semi-automatically segmented the cerebrum and CSF, cerebellum and brainstem from the T2-weighted MR images (CISS) of 17 newborn and 17 fetal subjects part of the MaRIAS study using an algorithm based on an Expectation-Maximization process combined with a prior relaxation scheme (AdaPT) (Cardoso et al., 2013).

We compared the estimated cerebrum weight for the newborn and fetal cohorts with the available autopsy weights. Both cohorts showed that MRI-derived brain weights were accurate. However, the results of the fetus cohort appeared more accurate, probably due to smaller variation among the subjects resulting from their smaller differences in age, shape, size or structure.

Disagreement between the MRI-derived and autopsy weights could be due to fluid loss. In the computation of the MRI brain weight, we attempted to exclude the contribution of the fluid based on the assumption that it partially leaks in the autopsy process and therefore is lost before weighing. However, some fluid might remain in the tissue, contributing to the autopsy weights. Further disagreement between the two values could also come from the cerebrum density value used from the literature, which may not be accurate for our subjects. The fixation process of the brain can also introduce errors in the conventional autopsy brain weight and cause disagreement with the MRI brain weight.

The average Dice overlap between the semi-automatic and manual segmentations showed good average agreement for all structures. The Dice score is affected by structure size (Dice, 1945), thus explaining the lower Dice scores in cerebellum and brainstem when compared to the cerebrum and CSF. In tissue segmentation, the choice of the atlas is extremely important. Having an atlas based on subjects that undergo similar post-mortem processes will improve the results. When compared with the manual segmentation, the fetus segmentation obtained using priors derived from the public atlas had lower Dice score values than those obtained using the newly created newborn atlas (Table 1 and Fig. 3). Although the contrast between the fetal brain and the newborn one, part of the same study, might be larger, the contrast between gray matter, white matter and CSF is much lower in both cohorts. This low difference could potentially explain the better fetal segmentation when using the created atlas over the publicly available one.

There are several limitations to the current study. Firstly, we used a relatively small subset of the MaRIAS cohort, which may restrict the generalizability of our conclusion. Secondly, the subjects that were part of this study had no major brain damage and thus we cannot state that the cerebrum estimation algorithm would be appropriate for those subjects of MaRIAS or other post-mortem studies who present lesions or cerebral hemorrhage. Furthermore, the subjects included in the study also had good resolution and contrast and no major imaging artifact. Although the estimation was not tested on post-mortem brains that present different pathologies or have lower quality images, the AdaPT segmentation algorithm might be able to successfully segment brain lesions because it has been shown to segment highly variable cases in neonates (Cardoso et al., 2013). Thirdly, as mentioned, the brain is separated into three parts during conventional autopsy: cerebrum, cerebellum and brainstem, but we were only provided with the cerebrum weights. If cerebellum or brainstem weights were also available we could further validate whether a good estimation can be made for all brain structures. Lastly, the main limitation of our study is that we were not able to separate the CSF from the cerebrum automatically, because of the brain decay after death and the relaxation rates, T1 and T2, increasing and converging to free water values (Thayyil et al., 2012). We attempted to separate the cerebrum/CSF by manually choosing thresholds. We report all CSF threshold values to ensure reproducibility and guidance for future studies. This issue might be solved in the future by using a post-mortem neonatal atlas, which should include a CSF class, created from manual segmentations of MR images of such subjects. Another solution to make the choice of the CSF threshold automatic might be creating an intensity histogram of the cerebrum-CSF segmentation. With sufficient further information, like post-mortem T2 relaxometry (Lally et al., 2014) brain maceration in fetuses and newborns could also be modeled and thus included as part of the segmentation process.

Nevertheless, post-mortem MRI can give a good estimation of the cerebrum weight for neonatal subjects, which is an important part of autopsy. The results of this section are notable, since they strengthen the idea of using MRI as an alternative to conventional autopsy, an alternative that can be more acceptable for parents and society at large. It also does not delay the funeral since it does not require a fixation process. To our knowledge this is the first paper to estimate brain weights from MR images of neonates and fetuses for the purpose of autopsy. The processing pipeline is reproducible since all the software used in this study is publicly available. We used the default registration parameters for all of the registration steps mentioned. Furthermore, the atlases used as prior information for segmentation are also freely available (Kuklisova-Murgasova et al., 2011) and the newborn post-mortem atlas created will be made available. We speculate that this availability will allow further research of post-mortem MRI of neonates and fetuses.

## 5 Conclusion

We segmented the cerebrum and CSF, cerebellum and brainstem of post-mortem newborn and fetal subjects of MaRIAS using atlas-based segmentation and an EM-based algorithm with relaxation priors. We validated the semi-automatic segmentation by computing the Dice overlap with manual segmentations. The study's aim was the comparison of semi-automatic segmentation results with the ones obtained during conventional autopsies. The excellent agreement of the segmentation-derived weights with the autopsy ones are of great importance, since it supports the use of post-mortem MRI, currently alongside other minimally invasive procedures, as a viable alternative for conventional autopsy of infants and fetuses.

## Figures and Tables

**Table 1:** Dice overlap of automatic segmentation with manual segmentation for each structure among each cohort.

	Cerebrum + CSF	Cerebellum	Brainstem
Newborn	0.991 ± 0.002	0.873 ± 0.028	0.819 ± 0.031
Fetus	0.992 ± 0.002	0.888 ± 0.016	0.815 ± 0.035
Fetus (*public atlas*)	0.984 ± 0.012	0.756 ± 0.185	0.676 ± 0.262

**Table 2:** Comparison between MRI weights, MRI manual weights and autopsy weights of the cerebrum for newborns. Density for cerebrum used to compute the weight from the MR volume 1.08 g/mL. The threshold values used to remove the CSF from the MR segmentation are also included.

MaRIAS no.	Gestational age [weeks]	Postnatal age [days]	CSF threshold value	MRI volume [mL]	MRI weight [g]	MRI manual weight [g]	Autopsy weight [g]
193	40	21	600	418.15	451.61	446.08	457.0
194	39	1	600	418.56	452.05	446.46	462.5
256	38	1	800	360.43	389.27	N/A	433.0
259	29	120	400	413.45	446.52	442.92	456.7
262	37	3	500	338.13	365.18	N/A	368.0
274	40	2	600	430.50	481.29	N/A	N/A
276	41	1	600	333.32	359.99	N/A	367.0
293	35	10	600	329.07	355.40	350.62	360.0
300	40	1	400	388.61	419.70	N/A	420.0
306	37	1	500	287.15	345.49	309.86	N/A
313	37	3	800	310.26	335.08	N/A	360.0
315	40	3	900	321.41	347.13	347.30	409.0
318	40	2	400	313.07	338.12	N/A	368.0
325	40	30	500	586.24	633.14	627.79	635.0
356	40	1	600	428.28	462.54	N/A	445.9
368	35	5	600	345.69	373.35	N/A	363.8
386	40	30	700	501.73	541.87	534.37	609.0

**Table 3:** Comparison between MRI weights, MRI manual weights and autopsy weights of the cerebrum for fetuses. Density for cerebrum used to compute the weight from the MR volume 1. 08 g/mL. The threshold values used to remove the CSF from the MR segmentation are also included.

MaRIAS no.	Gestational age		CSF threshold value	MRI volume [mL]	MRI weight [g]	MRI manual weight [g]	Autopsy weight [g]
	[weeks]	[days]					
121	39	0	700	361.74	390.67	390.40	388.00
149	34	2	700	267.79	289.22	N/A	N/A
196	32	2	800	160.66	173.52	172.59	181.00
210	38	6	600	354.74	383.12	N/A	391.00
214	37	2	550	298.07	321.92	N/A	328.00
230	41	6	450	370.73	400.39	N/A	401.00
246	32	6	530	208.06	224.70	225.54	223.70
282	30	5	500	146.96	158.72	N/A	N/A
299	44	0	300	408.43	441.11	438.00	447.00
330	35	6	800	264.72	285.90	N/A	292.00
339	36	2	800	339.31	366.45	365.05	N/A
343	34	1	350	247.69	267.50	N/A	275.00
379	38	5	290	357.13	385.70	N/A	386.13
384	39	4	300	366.63	395.96	N/A	389.80
388	38	0	390	439.41	474.56	469.56	N/A
391	33	0	550	213.39	230.46	231.59	226.00
396	29	0	700	113.70	122.80	122.22	128.00

## Footnotes

1 The publicly available neonate brain atlas can be found at: http://biomedic.doc.ic.ac.uk/brain-development/index.php?n=Main.Neonatal.

2 NiftySeg is an open source software available at http://sourceforge.net/projects/niftyseg/.

3 NiftyReg is an open source software available at http://sourceforge.net/projects/niftyreg/.
